# Predict lncRNA-drug associations based on graph neural network

**DOI:** 10.3389/fgene.2024.1388015

**Published:** 2024-04-26

**Authors:** Peng Xu, Chuchu Li, Jiaqi Yuan, Zhenshen Bao, Wenbin Liu

**Affiliations:** ^1^ Institute of Computational Science and Technology, Guangzhou University, Guangzhou, China; ^2^ School of Computer Science of Information Technology, Qiannan Normal University for Nationalities, Duyun, China; ^3^ College of Information Engineering, Taizhou University, Taizhou, Jiangsu, China; ^4^ Guangdong Provincial Key Laboratory of Artificial Intelligence in Medical Image Analysis and Application, Guangzhou, Guangdong, China

**Keywords:** lncRNA-drug association, graph attention networks, principal component analysis, drug discovery, link prediction

## Abstract

LncRNAs are an essential type of non-coding RNAs, which have been reported to be involved in various human pathological conditions. Increasing evidence suggests that drugs can regulate lncRNAs expression, which makes it possible to develop lncRNAs as therapeutic targets. Thus, developing *in-silico* methods to predict lncRNA-drug associations (LDAs) is a critical step for developing lncRNA-based therapies. In this study, we predict LDAs by using graph convolutional networks (GCN) and graph attention networks (GAT) based on lncRNA and drug similarity networks. Results show that our proposed method achieves good performance (average AUCs > 0.92) on five datasets. In addition, case studies and KEGG functional enrichment analysis further prove that the model can effectively identify novel LDAs. On the whole, this study provides a deep learning-based framework for predicting novel LDAs, which will accelerate the lncRNA-targeted drug development process.

## 1 Introduction

LncRNAs are a class of non-coding RNAs transcribed from DNA with a length of over 200 nucleotides (Ponting et al., 2009). They account for 70%–80% of all non-coding RNAs and play crucial regulatory roles in numerous cellular processes, including but not limited to transcription, splicing, translation, DNA repair, and regulation of genes. The quantity and biological importance of lncRNAs determine their widespread involvement in all physiological activities of living cells and the pathogenesis of human diseases, such as cancer, Parkinson’s disease, and cardiovascular disease (Riva et al., 2016; Schmitz et al., 2016; McCabe and Rasmussen, 2021). LncRNAs represent a new type of potential therapeutic targets, that can affect the diagnosis, treatment, and prognosis of diseases, and have attracted significant attention (Blokhin et al., 2018; Fernandes et al., 2019; Winkle et al., 2021).

Due to the key roles of lncRNAs in diseases, it is crucial to develop lncRNA-targeted drugs and technologies. This presents a significant opportunity for the treatment of lncRNA-related diseases and represents a new area for drug development (Sangeeth et al., 2022). Emerging studies have shown that small-molecular drugs can inhibit the proliferation of tumor cells or tumor stem cells by regulating the expression of lncRNAs, laying a crucial theoretical foundation for the advancement of lncRNA-targeted therapeutics (Liu et al., 2021). To develop lncRNA-targeted drugs, it is necessary to take three preparatory steps: elucidating the action mechanism of lncRNAs in diseases, analyzing their structural and functional pockets, and finding small-molecular drugs that can bind specifically to the pockets. One important aspect of this process is identifying associations between lncRNAs and drugs (Jiang et al., 2019; Chen Y. et al., 2021). Predicting lncRNA-drug associations (LDAs) not only facilitates the selection of potential drug candidates but also streamlines the drug discovery process, ultimately propelling the realization of efficacious lncRNA-targeted therapies and fostering advancements in precision medicine.

LDAs are mainly identified through biological experiments. For example, curcumin plays a crucial role in the treatment of various cancers by regulating lncRNAs (Patel et al., 2020). It inhibits the expression of lncRNA H19, and restores MEG3 levels via demethylation, thus enhancing the sensitivity of cancer cells to chemotherapeutic drugs (Zhang et al., 2017; Cai et al., 2021). Wang et al. (2020) confirmed that the oncogenic factor lncRNA CCAT2 was overexpressed in ovarian cancer, and calcitriol, the vitamin metabolite, can inhibit the proliferation, migration, and differentiation of ovarian cancer cells by inhibiting the expression of CCAT2. However, identifying LDAs based on biological experiments is time-consuming and costly, there is a need for efficient and accurate computational methods to predict potential LDAs, which can be further verified by biological experiments.

Jiang et al. (2019) identified LDAs based on the hypothesis that lncRNAs with similar sequences are often regulated by the same drug, and drugs with similar structures tend to regulate the same lncRNA. Wang et al. (2018) utilized Elastic Network (EN) regression to predict potential LDAs by integrating lncRNA expression profiles and drug response data in cancer cells. However, the limitation of these methods is that they heavily rely on specific features of the existing data, which may affect the prediction performance. Although predicting LDAs is receiving increasing attention, the relevant prediction methods are still relatively lacking.

At present, a wealth of computational methods has been accumulated for predicting small molecule drug-miRNA associations (Qu et al., 2019; Yin et al., 2019; Chen et al., 2020). Considering that both lncRNAs and miRNAs are non-coding RNAs involved in gene expression regulation and cellular functions, and share similar regulatory mechanisms (Yan and Bu, 2021), the methods for predicting drug-miRNA interactions hold significant implications for predicting LDAs. Zhao et al. (2020) developed a model using symmetric nonnegative matrix factorization and Kronecker regularized least squares to predict small molecule drug-miRNA associations. Chen et al. (2021) predicted small molecule drug-miRNA associations based on bounded nuclear norm regularization. Wang et al. (2022) presented an ensemble of kernel ridge regression-based method to identify potential small molecule-miRNA associations. Niu et al. (2023) employed a combination of GNNs and Convolutional neural networks (CNNs) to predicted small molecule drug-miRNA association.

Recently, due to the breakthroughs in deep learning and the huge improvements in computing power, models based on deep learning, particularly those employing GNNs, have been applied in multiple bioinformatics-related tasks, such as lncRNA-disease association prediction, and drug-target interaction prediction (Xuan et al., 2019; Chen et al., 2020; Kumar Shukla et al., 2020; Zhao et al., 2023). Yin et al. (2023) proposed a general framework using residual GCN and CNNs to predict drug-target interactions. Wang and Zhong (2022) proposed a method (gGATLDA) to identify potential lncRNA-disease associations based on graph attention networks (GAT). The success of the above GNNs-based methods can be attributed to the three main reasons: 1) biological correlations can be modeled naturally as graph structures, 2) GNNs have the advantage of capturing complex network relationships, 3) the introduction of attention mechanisms enables the model to focus locally on important nodes in the graph and effectively integrate node information on a global scale.

Therefore, based on the experiments validated LDAs dataset (D-lnc (Jiang et al., 2019)), we propose a GNNs-based framework to predict LDAs by referring to the gGATLDA method which was originally designed to predict lncRNA-disease associations. In this paper, we first extract the lncRNA-drug bipartite graph according to the LDAs matrix and obtain one-hop enclosing subgraphs of all lncRNA-drug pairs from the bipartite graph. Then, the feature vectors of lncRNA-pairs are constructed according to Gaussian interaction profile kernel lncRNA (drug) similarities. Finally, GCN learns lncRNA and drug node embeddings and obtain local spatial characteristics of nodes. GAT uses the attention mechanism to integrate the global information of the lncRNA-drug bipartite graph. Our model takes full advantage of GCN and GAT to predict novel LDAs. Results show that the method achieves high AUC and AUPR, and the ablation experiments show that our model performs better than GCN and GAT. The case studies on two drugs (Berberine and Panobinostat) and two lncRNAs (NEAT1 and MEG3) demonstrate the effectiveness of the model in predicting the potential LDAs. In the functional enrichment analysis, we further verified the validity of our predicted LDAs from the perspective of the relationship between the biological function of drugs and the enrichment pathway of lncRNA target genes. All these results suggest that the framework used in this study is an efficient method for predicting LDAs.

## 2 Materials and methods

### 2.1 Materials

Three benchmark datasets including the Gene Expression Omnibus (GEO) dataset, Connectivity Map (cMap) dataset, and Validated dataset (Jiang et al., 2019) are downloaded from http://www.jianglab.cn/D-lnc/index.jsp. The cMap dataset and GEO dataset were obtained by re-annotating microarray probes in cMap and GEO databases, respectively, and screening lncRNA differential expression data before and after drug therapy. The Validated dataset was created by searching experimentally verified drug modification of lncRNA expressions. We obtain three benchmark datasets (Dataset 1, Dataset 2, and Dataset 3) by removing the repeated LDAs of GEO dataset, cMap dataset, and Validated dataset respectively. As the number of lncRANs and drugs in three benchmark datasets is unbalanced, only when Dataset 1 and Dataset 2 are combined, the number of them is relatively balanced. Therefore, we combine Dataset 1 and Dataset 2 into Dataset 4, which is used as a training dataset in the case study to predict LDAs in Dataset 3 (see case study section). Dataset 5 is merged from Dataset 1, Dataset 2, and Dataset 3. Table 1 shows the detailed information of five datasets. We treat the known LDAs as positive samples and randomly select the negative samples with the same number of positive samples from the unknown LDAs.

**TABLE 1:** The detailed information of five datasets.

	LncRNAs	Drugs	Associations
Dataset 1	2360	115	28487
Dataset 2	129	1279	15804
Dataset 3	4691	48	4791
Dataset 4 (Dataset 1 + Dataset 2)	2431	1369	44262
Dataset 5 (Dataset 1 + Dataset 2 + Dataset 3)	6556	1400	49044

### 2.2 Methods

**FIGURE 1:**
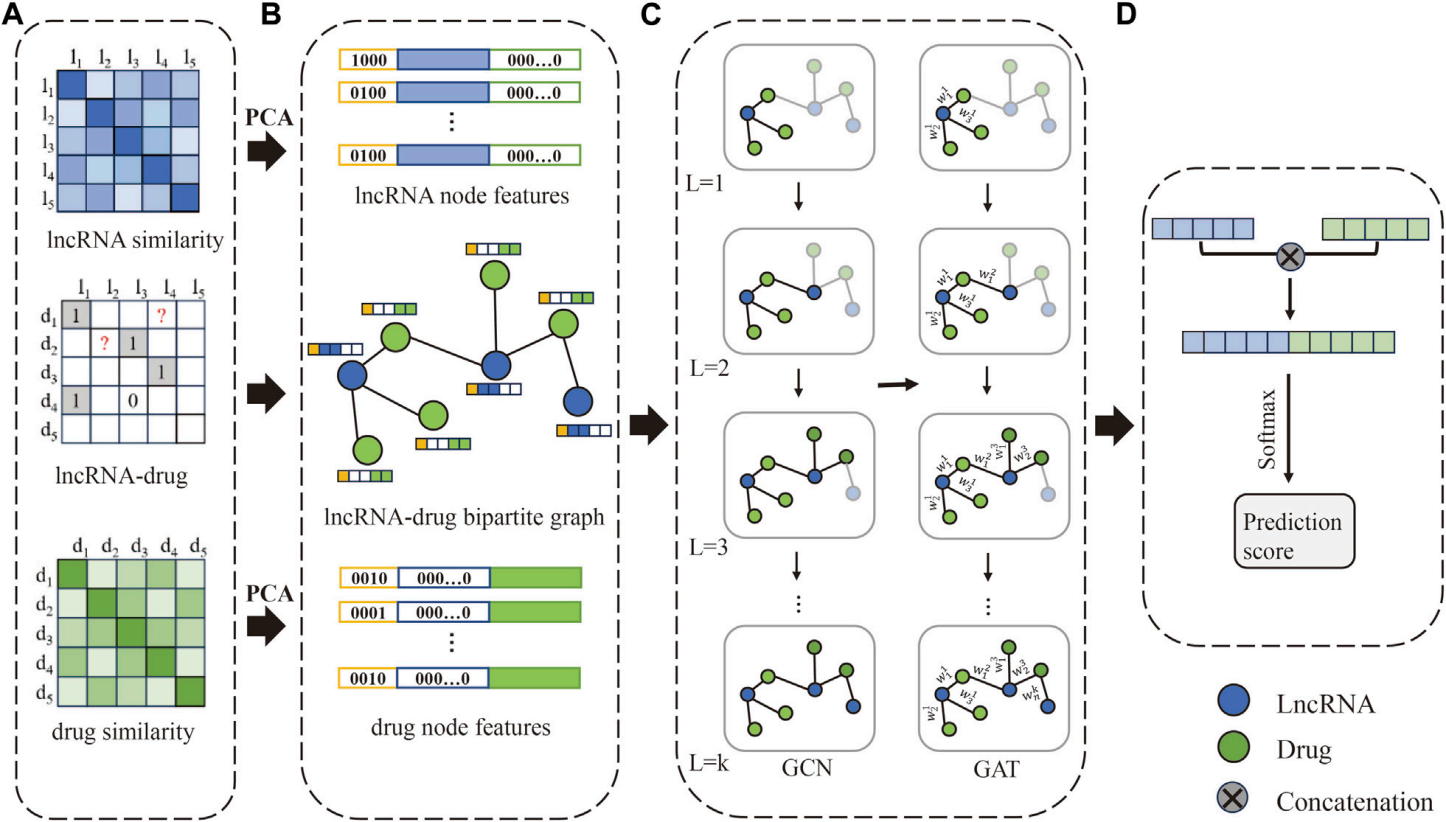
The flowchart of our method. **(A)** The LDAs matrix, lncRNA similarity matrix, and drug similarity matrix are constructed, respectively. **(B)** Obtain lncRNA-drug bipartite graph and one-hop subgraphs, and construct the initial feature vector of lncRNA and drug. **(C)** Extract feature representation of lncRNAs and drugs based on GCN and GAT. **(D)** Concatenate the lncRNA vector and drug vector to obtain a new vector, and the prediction score is obtained by Softmax.

The flowchart of the method is shown in Figure 1. Firstly, construct the lncRNA-drug association matrix, lncRNA similarity matrix, and drug similarity matrix. Secondly, construct one-hop enclosing subgraphs according to the lncRNA-drug bipartite graph, and obtain lncRNA node features and drug node features, respectively. Further, the one-hop enclosing subgraph of each lncRNA-drug pair and their feature vectors are input to the GNNs model. Finally, the lncRNA vector and drug vector are concatenated and processed by Softmax to obtain the prediction score.

#### 2.2.1 Constructing similarity matrices for lncRNAs and drugs

Because of the sparsity of the lncRNA-drug association matrix $LD\in R^{m\times n}$ , we calculate lncRNA similarity $LS\left(l_{i},l_{j}\right)$ and drug similarity $DS\left(d_{i},d_{j}\right)$ by the following Gaussian interaction profile kernel (GIP) (van Laarhoven et al., 2011; Yang and Li, 2021):

$$LS\left(l_{i},l_{j}\right)=\exp\left(-r_{l}{\left\|IP\left(l_{i}\right)\;-\;IP\left(l_{j}\right)\right\|}^{2}\right)$$ (1)

$$DS\left(d_{i},d_{j}\right)=\exp\left(-r_{d}{\left\|IP\left(d_{i}\right)\;-\;IP\left(d_{j}\right)\right\|}^{2}\right)$$ (2)

where $LS\in R^{m\times m}$ and $DS\in R^{n\times n}$ denote the lncRNA similarity matrix and drug similarity matrix, respectively. m and n represent the number of lncRNAs and drugs respectively. $IP\left(l_{i}\right)$ and $IP\left(d_{i}\right)$ are binary vectors, which represent the i th row and i th column of LD , respectively. If lncRNA $l_{i}$ is associated with drug $d_{j}$ , $LD\left(i,j\right)=1$ , otherwise $LD\left(i,j\right)=0$ . $r_{l}$ and $r_{d}$ are used to adjust the kernel bandwidth, which are calculated as followed:

$$r_{l}=1/\left(\frac{1}{m}\sum_{i=1}^{m}\left\|IP\left(l_{i}\right)\right.{\|}^{2}\right)$$ (3)

$$r_{d}=1/\left(\frac{1}{n}{\sum_{i=1}^{n}\left\|IP\left(d_{i}\right)\right\|}^{2}\right)$$ (4)

#### 2.2.2 Extracting one-hop enclosing subgraph

The bipartite graph G is constructed from the matrix LD , where each known lncRNA-drug pair corresponds to an edge connecting the lncRNA $l_{i}$ and drug $d_{j}$ , for unknown LDAs, there are no edges between $l_{i}$ and $d_{j}$ . The one-hop enclosing subgraph $G1\left(V_{1},E_{1}\right)$ of each lncRNA-drug pair $\left(l_{i},d_{j}\right)$ can be defined as following: $V_{1}$ is the set of nodes containing one-hop neighbor nodes of $l_{i}$ , one-hop neighbor nodes of $d_{j}$ , as well as node $l_{i}$ and node $d_{j}$ , and $E_{1}$ is edge set. Each node in subgraphs can be labeled to distinguish its role (Zhang and Chen, 2020). We use 0 and 1 to label target lncRNA node and target drug node, respectively, and use 2i and 2i+1 to label the one-hop neighbor nodes of $l_{i}$ and the one-hop neighbor nodes of $d_{j}$ , respectively, where i represents the order of neighbor nodes, and it is set to 1 according to gGATLDA (Wang and Zhong, 2022).

#### 2.2.3 Constructing and denoising original feature vectors

The original lncRNA and drug feature vectors are constructed from lncRNA similarity matrix and drug similarity matrix, respectively. However, due to the high dimension of original features and the sparsity of lncRNA (drug) similarity matrix, we employ principal component analysis (PCA) for dimension reduction. PCA is a classical, efficient, and unsupervised feature selection method, which can not only retain as much feature information as possible while reducing the feature dimension but also greatly reduce the training time of the model. Assume the original lncRNA and drug feature vector $f_{l}^{0}=\left[f_{l1}^{0},f_{l2}^{0},f_{l3}^{0},...,f_{lm}^{0}\right]$ and $f_{d}^{0}=\left[f_{d1}^{0},f_{d2}^{0},f_{d3}^{0},...,f_{dn}^{0}\right]$ , respectively. After performing PCA, we obtain the feature vectors $f_{l}^{\prime }=\left[f_{l1}^{\prime },f_{l2}^{\prime },f_{l3}^{\prime },...,f_{la}^{\prime }\right]$ and $f_{d}^{\prime }=\left[f_{d1}^{\prime },f_{d2}^{\prime },f_{d3}^{\prime },...,f_{db}^{\prime }\right]$ , where a and b denote the feature vector dimension of lncRNAs and drugs, respectively. Since a and b may not be equal, in order to ensure that the input feature dimensions of each node are the same, we take the sum of 4+a+b as the feature dimensions of the nodes, where the first 4 dimensions represent the one-hot encoding of the node labels to distinguish the roles of different nodes. The extra b dimensions of lncRNA node features and a dimensions of drug node feature are filled with 0 values. We construct the lncRNA feature matrix $F_{lnc}=R^{m\times \left(4+a+b\right)}$ and the drug feature matrix $F_{drug}=R^{m\times \left(4+a+b\right)}$ . The feature vector of lncRNA l is $f_{l}=\left[p_{1},p_{2},p_{3},p_{4},f_{l1}^{\prime },f_{l2}^{\prime },f_{l3}^{\prime },...,f_{la}^{\prime },0,0,0,...,0\right]$ , and $p_{j}$ $\left(1\leq j\leq 4\right)$ represents the one-hot encoding of the node label, distinguishing different roles. Similarly, the feature vector of a drug d is $f_{d}=\left[p_{1},p_{2},p_{3},p_{4},0,0,0,...,0,f_{d1}^{\prime },f_{d2}^{\prime },f_{d3}^{\prime },...,f_{db}^{\prime }\right]$ .

#### 2.2.4 The model based on GCN and GAT

GNNs are a class of data models for processing graph structures that utilize a message-passing mechanism to update the node embeddings. GCN and GAT are two specific GNN models whose core idea is to update node embeddings by aggregating neighbor nodes’ information. The difference is that GAT introduces an attention mechanism during the message-passing process, which can adaptively assign different weights to different nodes, allowing for more flexibility in capturing relationships between nodes.

In the model, the GCN layer is employed to update the features of lncRNA and drug nodes. The feature representation of node i in the k+1 layer is presented as follows:

$$h_{i}^{\left(k+1\right)}=\sigma \left(\sum_{j\in N\left(i\right)\cup \left[i\right]}\frac{1}{\sqrt{d\left(i\right)}\cdot \sqrt{d\left(j\right)}}\left(h_{j}^{\left(k\right)}W^{\left(k+1\right)}\right)\right)$$ (5)

where $h_{j}^{\left(k\right)}$ represents the feature vector of node j in layer k $\left(k=0,1,2,3,...,n\right)$ , $d\left(j\right)$ denotes the degree of node j , $N\left(i\right)$ represents the set containing all neighbors of node i , and $W^{\left(k+1\right)}$ denotes the parameter matrix to be learned in the k+1 GCN layer.

To get the weights between different nodes, GAT introduces the attention coefficient. The attention coefficient between node i and node j is calculated as follows:

$$e_{ij}=\sigma \left(a\left(W^{\left(k\right)}h_{i}^{\left(k\right)},W^{\left(k\right)}h_{j}^{\left(k\right)}\right)\right)$$ (6)

where a represents a shared attention mechanism to calculate the attention coefficient, and σ represents the LeakyReLU activation function.

To compare the attention coefficient between different nodes, the normalized attention coefficient $\alpha_{ij}$ is calculated as follows:

$$\alpha_{ij}=softmax\left(e_{ij}\right)=\frac{\exp\left(e_{ij}\right)}{\sum_{m\in N_{i}}\exp\left(e_{im}\right)}$$ (7)

After obtaining the attention coefficients between node i and its neighbor nodes, we obtain the final representation $h_{i}^{\left(k+1\right)}$ by taking a weighted summation of its neighbor nodes, and it is calculated as follows:

$$h_{i}^{\left(k+1\right)}=\sigma \left(\sum_{j\in N_{i}}\alpha_{ij}W^{\left(k\right)}h_{j}^{\left(k+1\right)}\right)$$ (8)

where σ represents ELU activation function.

#### 2.2.5 Prediction score

For the final output of the last GAT layer, the vector representations of target lncRNA and target drug are concatenated:

$$f_{\left(l_{i},d_{j}\right)}=concat\left(h_{l_{i}},h_{d_{j}}\right)$$ (9)

where $h_{l_{i}}$ and $h_{d_{j}}$ denote the final feature representation of the lncRNA $l_{i}$ and $d_{j}$ , respectively. The purpose of concatenating the feature vectors of target lncRNA and target drug is to integrate the feature information of lncRNA and drug node pairs to form a richer feature representation and to reduce information loss to a certain extent. This can help the model better understand the relationship between lncRNAs and drugs and improve the accuracy of prediction.

Finally, for the representation $f_{\left(l_{i},d_{j}\right)}$ , we use Softmax as an activation function to obtain the prediction score $y^{\prime }\left(l_{i},d_{j}\right)$ :

$$y_{\left(l_{i},d_{j}\right)}^{\prime }=Softmax\left(f_{\left(l_{i},d_{j}\right)}\right)=\frac{e^{f_{\left(l_{i},d_{j}\right)}}}{\sum_{j=1}^{n}e^{f_{\left(l_{i},d_{j}\right)}}}$$ (10)

The binary cross-entropy loss function is used to train the weight $W^{\left(k\right)}$ :

$$Loss=-y_{\left(l_{i},d_{j}\right)}\log\left({y^{\prime }}_{\left(l_{i},d_{j}\right)}\right)+\left(1\;-\;y_{\left(l_{i},d_{j}\right)}\right)\log\left(1\;-\;{y^{\prime }}_{\left(l_{i},d_{j}\right)}\right)$$ (11)

where $y_{\left(l_{i},d_{j}\right)}$ represents the real label.

### 2.3 Evaluation criteria

In this study, we evaluate the performance of the model by means of AUC (Area Under Curve), AUPR (Area Under the Precision-Recall curve), precision, accuracy, F1-Score, and recall. AUC means the area under the Receiver Operating Characteristic (ROC) curve, which is plotted by the true positive rate (TPR) and the false positive rate (FPR) at different thresholds. The TPR and FPR are calculated as follows:

$$FPR=\frac{FP}{FP+TN}$$ (12)

$$TPR=\frac{TP}{TP+FN}$$ (13)

where TP and TN are the numbers of correctly identified positive and negative samples respectively. FP and FN are the numbers of misidentified positive and negative samples, respectively.

In addition, the evaluation metrics including precision, recall, F1-score, and accuracy are calculated as follows:

$$precision=\frac{TP}{TP+FP}$$ (14)

$$recall=\frac{TP}{TP+FN}$$ (15)

$$F1-score=\frac{2\times precision\times recall}{precision+recall}$$ (16)

$$Accuracy=\frac{TP+TN}{TP+TN+FP+FN}$$ (17)

## 3 Results

In this section, firstly, we select the appropriate parameters of the model through parameter optimization. Secondly, we show the experimental results of six evaluation metrics on five datasets and conduct ablation experiments on five datasets. Thirdly, case studies are conducted on two drugs and two lncRNAs, which aim to validate the ability of the method to predict potential LDAs. Finally, we performed the KEGG functional analysis based on the results of case studies to further verify the validity of the predicted LDAs, especially for those that are unconfirmed in the case study.

### 3.1 Parameter optimization

**FIGURE 2:**
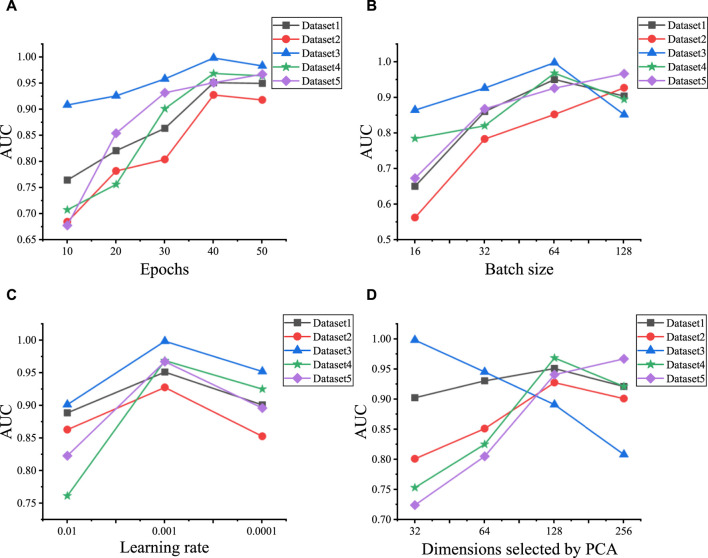
Hyperparameter optimization results for five datasets. **(A)** The AUC values under the different epochs on five datasets. **(B)** The AUC values under the different batch sizes on five datasets. **(C)** The AUC values under the different learning rates on five datasets. **(D)** The AUC values under the different dimensions selected by PCA on five datasets.

We initially explore the influence of various hyperparameter combinations on the performance of predicting LDAs across five datasets. These hyperparameters include epochs, batch size, learning rate, the initial feature vector dimensionality selected by PCA, and the layers of GCN and GAT, respectively. We utilize grid search to tune these six hyperparameters. The epochs range extends from 10 to 50, incremented by 10. Batch size is selected from the set {16, 32, 64, 128}, learning rate is chosen from {0.1, 0.001, 0.0001}, and the initial feature vector dimensionality selected by PCA ranges from {32, 64, 128, 256}. The experimental results are shown in Figure 2, the optimal model performance is attained when a particular combination of hyperparameters is used on Dataset 1 to Dataset 5. Specifically, epochs are set to {40, 40, 40, 40, 50}, batch sizes to {64, 128, 64, 64, 128}, and learning rate uniformly to 0.001. Additionally, the number of initial PCA-selected feature dimensions is set to {128, 128, 32, 128, 256} for each dataset.

In addition, the selection of model structure is crucial for prediction performance. Therefore, we also investigate the impact of the two main hyperparameters, the number of GCN and GAT layers on the model in Dataset 1, Dataset 2, and Dataset 3. Specifically, we select layer numbers ranging from 1 to 3 for both GCN and GAT. The increase in the number of GNN layers means aggregating features from higher-order neighbor nodes, but it may lead to the loss of local structure, resulting in overfitting and decreased prediction performance. For instance, as shown in Table 2, in Dataset 1, increasing the GCN layer from 1 to 2 improves performance, but further increasing it to 3 leads to a decrease in performance. It is worth noting that the model achieves the best performance across all three datasets when the model structure consists of 1 GCN layer and 3 GAT layers. Therefore, we choose 1 GCN layer and 3 GAT layers as our model architecture.

**TABLE 2:** The performance using different numbers of GCN and GAT layers on Dataset 1, Dataset 2, and Dataset 3.

		GAT×1		GAT×2		GAT×3	
	Datasets	AUC	AUPR	AUC	AUPR	AUC	AUPR
GCN×1	Dataset 1	0.8640	0.8463	0.9434	0.9059	**0.9514**	**0.9346**
	Dataset 2	0.7924	0.7733	0.8958	0.8575	**0.9280**	**0.9271**
	Dataset 3	0.9516	0.9215	0.9845	0.9651	**0.9986**	**0.9800**
GCN×2	Dataset 1	0.8990	0.8925	0.9101	0.8946	0.9111	0.8746
	Dataset 2	0.7941	0.7732	0.7783	0.7395	0.7965	0.7648
	Dataset 3	0.9542	0.9056	0.9158	0.8623	0.8954	0.8547
GCN×3	Dataset 1	0.8898	0.8766	0.8643	0.8019	0.8717	0.8856
	Dataset 2	0.7625	0.7722	0.7865	0.7581	0.7381	0.7052
	Dataset 3	0.9485	0.9156	0.9284	0.8956	0.9051	0.8451

The best performance on three datasets is highlighted in bold.

### 3.2 Prediction performance of the model

To avoid potential random bias, we repeat the five-cross-validation process 100 times and average the performance metrics to derive the final results. The results are shown in Table 3, the AUC and AUPR are higher than 0.92 on both the benchmark datasets and combined datasets. It is noteworthy that the overall performance of the model is superior on the combined datasets compared to the benchmark datasets. For instance, among the benchmark datasets, the model on Dataset 1 and Dataset 2 achieves the AUC of 0.9514 and 0.9280, respectively. However, the model’s AUC on Dataset 4 (Dataset 1+Dataset 2) is 0.9690, which surpassed that of its constituent subsets. This improvement of performance on the combined dataset can be attributed to the more balanced relationship between the quantities of lncRNAs and drugs and the more trainable data in this dataset. However, on the contrary, the performance of benchmark Dataset 3 is better than that of the combined datasets. This discrepancy can be attributed to the presence of 4791 LDAs involving 4691 lncRNAs and 48 drugs in Dataset 3. Notably, the average degree size of each drug is 99, implying that one drug corresponds to multiple lncRNA information, enabling the model to extract more complex features from abundant lncRNA information. Consequently, it facilitates a more accurate capture of the relationship between drugs and lncRNAs. Overall, these observations indicate that the proposed method has an excellent performance on the five datasets.

**TABLE 3:** The performance of the method on five datasets.

	Benchmark datasets			Combined datasets	
	Dataset 1	Dataset 2	Dataset 3	Dataset 4	Dataset 5
AUC	0.9514 ± 0.0027	0.9280 ± 0.0058	0.9986 ± 0.0013	0.9690 ± 0.0143	0.9675 ± 0.0133
AUPR	0.9346 ± 0.0063	0.9271 ± 0.0078	0.9800 ± 0.0835	0.9623 ± 0.0170	0.9763 ± 0.0318
Recall	0.9420 ± 0.0183	0.9487 ± 0.0140	0.9372 ± 0.0036	0.9838 ± 0.0194	0.9560 ± 0.0120
Precision	0.7213 ± 0.0114	0.7328 ± 0.0080	0.9507 ± 0.0028	0.9411 ± 0.0268	0.9723 ± 0.0180
Accuracy	0.8057 ± 0.0052	0.7485 ± 0.0100	0.9094 ± 0.0029	0.9513 ± 0.0221	0.9615 ± 0.0130
F1-Score	0.8375 ± 0.0064	0.7918 ± 0.0101	0.9799 ± 0.0030	0.9620 ± 0.0209	0.9723 ± 0.0117

### 3.3 Ablation experiment

**FIGURE 3:**
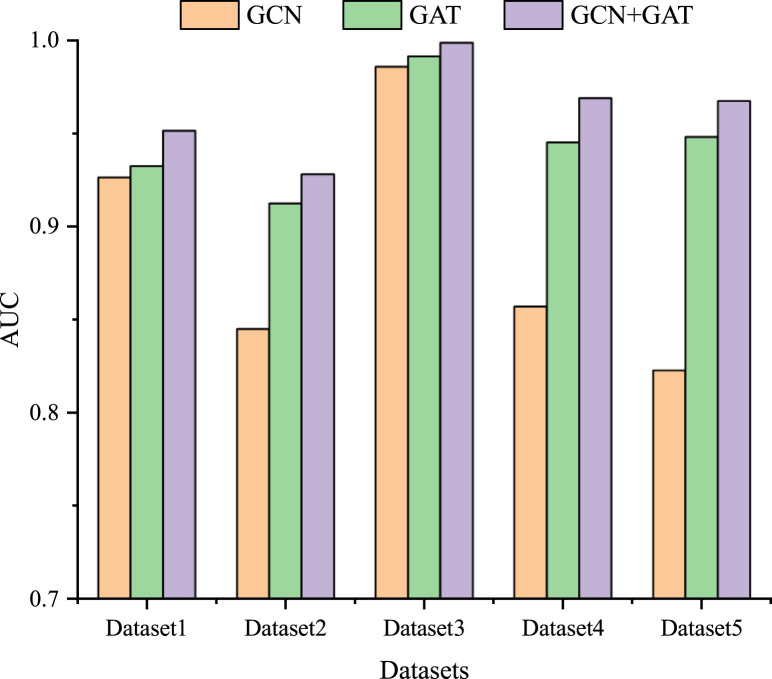
The results of the ablation experiment on five datasets.

To further study the influence of GCN and GAT on the model performance, we also conduct ablation experiments on five datasets. Specifically, we individually use the GCN and GAT modules, as well as their combined module for LDA prediction. As shown in Figure 3, among the three modules, the combined GCN and GAT modules obtain the optimal performance across all five datasets, followed by the GAT module, with the GCN module exhibiting the poorest performance. GCN module learns the feature representation of lncRNA and drug nodes by aggregating their neighbor information, which enables GCN to capture the spatial local structure of nodes. GAT module introduces an attention mechanism that allows the model to dynamically assign weights and integrate the global information of the lncRNA-drug bipartite graph, rather than just the neighbors of the lncRNA and drug nodes. Therefore, combining GCN and GAT modules enables the model to take full advantage of their strengths, complement each other, and further improve the prediction performance.

### 3.4 Case study

#### 3.4.1 Predicting potential lncRNAs and drugs

In this section, we conduct a case study to further demonstrate the performance of the proposed method, we predict LDAs for two drugs (Berberine and Panobinostat) and two lncRNAs (NEAT1 and MEG3), respectively. First, we predict two drug-related lncRNAs using all known LDAs in Dataset 4 excluding those in Dataset 3 as the training data. Dataset 3 is used as the ground truth to test the predicted LDAs. Second, given that Dataset 1 contains more lncRNA information compared to drugs, two lncRNA-related drugs are predicted by employing all known LDAs in Dataset 1 except for those in Dataset 2 as the training data. Dataset 2 is the ground truth to test the predicted drugs. The top 10 predicted LDAs are ranked according to their prediction scores, among those, for any associations not shown in the test datasets (Dataset 3 and Dataset 2), we manually search relevant literature in PubMed to provide supporting evidence.

**FIGURE 4:**
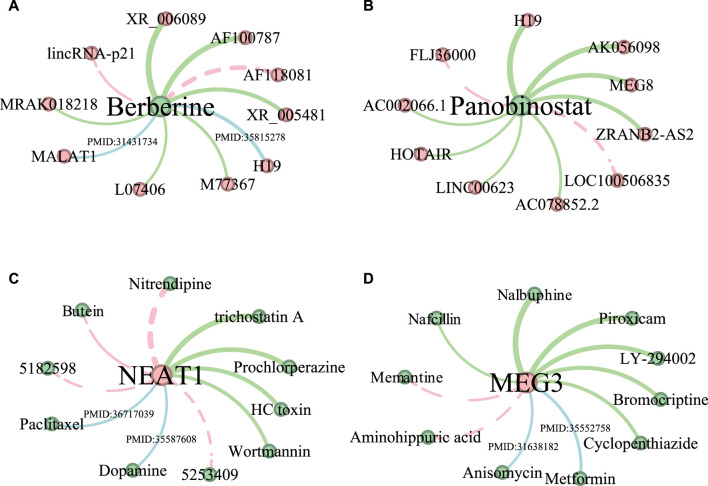
The predictive results of the top 10 lncRNAs associated with drugs Berberine **(A)** and Panobinostat **(B)**, and the top 10 drugs related to lncRNA NEAT1 **(C)** and MEG3 **(D)**. LncRNAs are represented by red nodes, while drugs are denoted as green nodes. The thickness of the edge indicates the predicted ranking, and the thicker the edge, the higher the ranking. Moreover, the LDAs found in test datasets are shown in green edges. Associations supported by existing literature are shown in blue edges, accompanied by their corresponding PMID references. Unconfirmed associations are represented by red dotted edges.

Figure 4 shows the top 10 predicted LDAs for the two drugs and two lncRNAs where the line width indicates the magnitude of the association score and the green lines indicate the confirmed LDAs in the test datasets. The blue lines indicate those LDAs that are not confirmed by the test dataset but have literature support. Generally, all the confirmed associations have large predicted scores. The red dotted lines represent LDAs having no support indication up to now. As shown in Figure 4A, 8 out of 10 predicted lncRNA-Berberine associations are validated, among which lncRNA “MALAT1” and “H19” are confirmed in the literature. Cao et al. (2020) demonstrated that lncRNA MALAT1 in cerebral ischemia was significantly reduced after treatment with the drug Berberine, highlighting its anti-inflammatory effects in mice after MCAO surgery. Song et al. (2022) identified lncRNA H19 as a potential key regulatory lncRNA of Berberine. Among the top 10 lncRNAs predicted associated with Panobinostat (Figure 4B), 8 lncRNAs are confirmed in test datasets. And for lncRNA NEAT1, 6 out of 10 predicted drugs related to NEAT1 are verified (Figure 4C). And 8 predicted drug-MEG3 associations have evidence in test datasets and literature (Figure 4D).

#### 3.4.2 Functional enrichment analysis

Since one lncRNA may regulate the expression of multiple downstream genes, therefore intervening with one lncRNA may involve a variety of biological functions. In addition, a drug may target a certain lncRNA to alter its expression, thereby regulating the expression of lncRNA target genes or modifying the activity of signaling pathways to realize its biological functions. For instance, Guo et al. (2016) showed that aspirin could significantly induce the expression of lncRNA OLA1P2 in human colorectal cancer, thereby affecting the activity of the STAT3 signaling pathway. To gain insights into the functional implications of the drugs and lncRNAs of concern, we conduct functional enrichment analysis on their related genes by an online tool DAVID (Sherman et al., 2022), which is widely used for functional annotation and enrichment analysis of gene lists.

**FIGURE 5:**
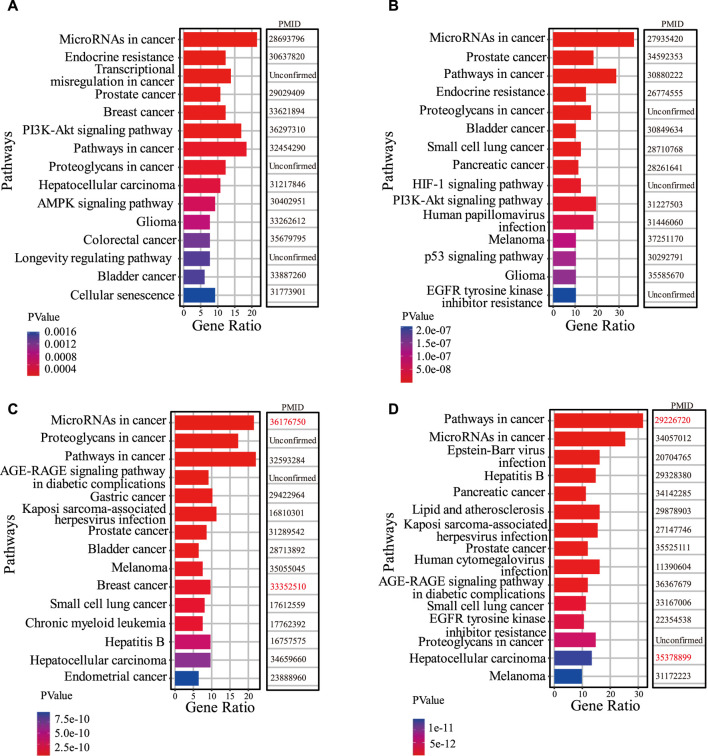
The results of functional analysis. **(A,B)** represent the top 15 KEGG pathways of the top 10 predicted Berberine-related lncRNA target genes and Panobinostat-related lncRNA target genes, respectively. **(C,D)** show the top 15 KEGG pathways of NEAT1 target genes and MEG3 target genes, respectively. The PMID of references are listed to confirm the interactions between pathways and known biological functions of drugs. The PMIDs marked in red represent literature confirming associations between KEGG pathways and the biological functions of drugs, but these drugs in Figures 4C, D are unconfirmed associated with lncRNA NEAT1 and MEG3.

Based on the results of the first part of the case study, firstly, we perform functional enrichment analysis on the target genes of lncRNAs predicted associated with two drugs (Berberine and Panobinostat). We search the literature demonstrating that drug functions are related to the enrichment pathways of lncRNA target genes. In Figure 5A, among the top 15 KEGG pathways of Berberine-related lncRNA target genes, 12 have been confirmed associated with the existing functions of Berberine. For example, Ayati et al. (2017) demonstrated that Berberine can play an anticancer role by regulating the expression of oncomiRs and tumor-suppressive miRs in various cancer cells (Hepatocellular carcinoma, gastric cancer, ovarian cancer, colorectal cancer). Okuno et al. (2022) evidenced that Berberine overcomes gemcitabine-related chemical resistance by modulating rap1/PI3K-akt signaling in pancreatic ductal adenocarcinoma. In Figure 5B, there are 12 KEGG pathways of predicted Panobinostat-related lncRNA target genes that are found to be associated with the functions of Panobinostat. Lee et al. (2017) demonstrated that Panobinostat overcame resistance to gefitinib in KRAS-mutant/EGFR wild-type non-small-cell lung cancer by targeting TAZ. Studies have also shown that Panobinostat can restore the sensitivity of endocrine-resistant and triple-negative breast cancer cell lines to estrogen receptors (Tan et al., 2016).

Further, the functional enrichment analysis is also conducted on the target genes of two lncRNAs (NEAT1 and MEG3). Figures 5C, D show the top 15 KEGG pathways of lncRNA NEAT1 and MEG3, respectively, among which 13 pathways are found associated with the known functions of predicted NEAT1-related drugs. Regarding the MEG3 KEGG pathways, 14 pathways are associated with the established functions of predicted MEG3-related drugs.

Among all KEGG pathways in Figures 5A–D, there are two common pathways, “MicroRNAs in cancer” and “pathways in cancer,” which are closely related to the occurrence and development of cancer. This demonstrates that lncRNAs, such as NEAT1 and MEG3, and lncRNAs associated with the drugs Berberine and Panobinostat, are widely involved in human pathogenesis. The biological function of the drugs Berberine and Panobinostat is related to the two common pathways, indicating that they can inhibit the proliferation of cancer cells by regulating the expression of lncRNAs’ target genes or changing the activity of the pathways. For example, Wang and Zhang (2018) showed that Berberine inhibits the proliferation and metastasis of endometrial cancer cells by inhibiting the expression of miR-101 target gene COX-2. LncRNA PINT is significantly downregulated in acute lymphoblastic leukemia (ALL), and drugs Panobinostat and Curcumin can reduce the proliferation of ALL cells by inducing the expression of PINT (Garitano-Trojaola et al., 2018). The results of functional enrichment analysis validated the importance of LDAs prediction for discovering potential lncRNA-targeted drugs to treat diseases.

## 4 Discussion

Predicting LDAs is beneficial for understanding the mechanism of drug-targeting lncRNAs to treat diseases at the lncRNA level, accelerating drug discovery and facilitating the development of targeted therapies. However, the identification of LDAs by traditional biological experiments has the disadvantages of high cost, long cycle, and low efficiency. Therefore, it is necessary to develop efficient computational methods to identify potential LDAS.

This study proposes a method based on GCN and GAT to predict potential LDAs. The results of five-cross-validation experiment on the five datasets show that our method achieves an excellent ability for LDA prediction. However, the performance of our model varies across the five datasets, mainly due to the following reasons: 1) the number of known LDAs in each dataset is different. Generally speaking, the more known LDA samples that can be trained on the model, the stronger the generalization ability of the model and the better the prediction performance. For example, our model performs better overall on combined Dataset 4 and Dataset 5 than on benchmark Dataset 1 and Dataset 2 (see Table 3). 2) the number distribution of lncRNAs and drugs may lead to a difference in the model’s predictive performance. For instance, although the number of lncRNAs and drugs in Dataset 3 is extremely unbalanced, the performance of the model on Dataset 3 is better than that in Dataset 4 (see Tables 1, 3).

In the case study, although some predicted LDAs could not be found in the test datasets, we verify the associations by reviewing the literature. Dong et al. (2022) found that NEAT1 promotes apoptosis and autophagy in Parkinson’s disease (PD) and inhibits the reproduction of dopaminergic neurons by targeting miR-107-5p (see Figure 4C). Wei et al. (2023) demonstrated that lncRNA NEAT1 can induce paclitaxel resistance in the breast cancer tumor microenvironment (see Figure 4C). Liu et al. (2022) revealed that Metformin plays a therapeutic role in endometrial hyperplasia by regulating the lncRNA MEG3/miR-233/GLUT4 signaling pathway (see Figure 4D). Ye et al. (2019) showed that Anisomycin inhibits angiogenesis, proliferation, and invasion of ovarian cancer cells by attenuating the molecular sponge effect of the lncRNA-MEG3/miR-421/PDGFRA axis (see Figure 4D).

For the LDAs that have not been verified in the case study, the functional analysis further elucidates their potential associations from the aspect of the relationship between the biological functions of drugs and the target gene pathways of lncRNAs. In particular, although no evidence is found for the drugs Butein and Bisphenol A (see Figure 4C) to be directly associated with lncRNA NEAT1, we find literature in which they are related to the pathways. For instance, Cioce et al. (2022) revealed that Butein weakened the pro-tumorigenic features of malignant pleural mesothelioma (MPM) via the miR-185-5p-TWIST1 axis. Deng et al. (2021) demonstrated that Bisphenol A promoted the proliferation of breast cancer cells by inhibiting miR-381-3p expression. Similarly, for the unconfirmed drugs Memantine and Aminohippuric acid associated with MEG3 (see Figure 4D), Memantine was found to induce apoptosis in prostate cancer cells and inhibit cell cycle progression (Albayrak et al., 2018), and Aminohippuric acid was identified as a gene-targeted therapy for ACBD4 in hepatocellular carcinoma (Huang et al., 2022).

Overall, this study demonstrates an efficient method to identify LDAs and provides an important basis for targeted therapy. To the best of our knowledge, this study is the first application of a deep learning-based model for inferring LDAs. Although our proposed method is used for predicting LDAs, it can also be extended to predict other association types, such as drug-drug interactions and drug-target interactions.

## 5 Conclusion

In this paper, we propose a model to identify potential LDAs, by integrating lncRNA and drug similarities after PCA denoising as attributes of nodes in the lncRNA-drug pair subgraphs. Leveraging the inherent graph structures of LDA network and similarity networks, GCN and GAT are used on each subgraph, allowing the model to selectively focus on important local information and integrate global information in the graph. The ablation experiments and cross-validation experiments on five datasets show that the method has good performance in LDA prediction. Furthermore, the case studies and functional enrichment analysis indicate the ability of the method to predict potential LDAs.

Although the model demonstrates great performance in predicting LDAs, it still has room for improvement. In the future, firstly, we still need to collect large-scale, high-quality datasets, which is crucial to improve the performance of LDA prediction. Secondly, our current model has not yet considered the drug structure feature representation and the lncRNA sequence feature representation. We will integrate them as lncRNA feature vectors and drug feature vectors in the future. Finally, we plan to develop more efficient deep learning methods (Lin et al., 2023) based on more types of association networks (Xu et al., 2020; Xu et al., 2022) to improve the prediction performance of LDAs.

## Data Availability

The original contributions presented in the study are included in the article/supplementary material, further inquiries can be directed to the corresponding authors. The code resource of this paper can be freely downloaded from https://github.com/LiChuchu123/LDA-based-GAT.
